# Release Profiles of Carvacrol or Chlorhexidine of PLA/Graphene Nanoplatelets Membranes Prepared Using Electrospinning and Solution Blow Spinning: A Comparative Study

**DOI:** 10.3390/molecules28041967

**Published:** 2023-02-19

**Authors:** Roberto Scaffaro, Luca Settanni, Emmanuel Fortunato Gulino

**Affiliations:** 1 Department of Engineering, University of Palermo, Viale delle Scienze, Ed. 6, 90128 Palermo, PA, Italy; 2 Department of Agricultural, Food and Forestry Sciences, University of Palermo, Viale delle Scienze, Ed. 5, 90128 Palermo, PA, Italy

**Keywords:** electrospinning, solution blow spinning, nanofibrous membranes, controlled release, carvacrol, chlorhexidine, graphene nanoplatelets, antimicrobial activity

## Abstract

Nanofibrous membranes are often the core components used to produce devices for a controlled release and are frequently prepared by electrospinning (ES). However, ES requires high production times and costs and is not easy to scale. Recently, solution blow spinning (SBS) has been proposed as an alternative technique for the production of nanofibrous membranes. In this study, a comparison between these two techniques is proposed. Poly (lactic acid)-based nanofibrous membranes were produced by electrospinning (ES) and solution blow spinning (SBS) in order to evaluate the different effect of liquid (carvacrol, CRV) or solid (chlorhexidine, CHX) molecules addition on the morphology, structural properties, and release behavior. The outcomes revealed that both ES and SBS nanofibrous mat allowed for obtaining a controlled release up to 500 h. In detail, the lower wettability of the SBS system allowed for slowing down the CRV release kinetics, compared to the one obtained for ES membranes. On the contrary, with SBS, a faster CHX release can be obtained due to its more hydrophilic behavior. Further, the addition of graphene nanoplatelets (GNP) led to a decrease in wettability and allowed for a slowing down of the release kinetics in the whole of the systems.

## 1. Introduction

Over the last years, controlled drug delivery systems have attracted much attention due to their advantages in efficacy, safety, and patient convenience [1,2,3,4]. Controlled release systems could be fabricated, using different production techniques in many forms, including 3D printed tablets, injectable microspheres or implants, and nanofibers mats [5,6,7,8]. These latter, in particular, are very promising for producing innovative drug delivery systems. Nanofibrous membranes, in fact, are characterized by a high specific surface area, high porosity, as well as tunable mechanical properties and morphology. Numerous nanofibrous systems were effectively used for achieving these results [9,10,11,12,13,14].

Electrospinning (ES) is one the most frequently reported technique to prepare nanofibers mats [12,15,16]. It has been recognized as promising technique to prepare membranes for controlled drug release [17]. ES easily allows to produce uniform and aligned fibers with homogeneous diameters that can form membranes with high and interconnective porosity. On the other hand, ES displays several disadvantages that have prevented industrial scalability such as a high production time, high energy consumption, and operator safety hazards [18,19,20]. In the scientific literature, several methods have been reported that allow for producing fibers, namely melt blowing, wet spinning, self-assembly, direct drawing, and phase separation [5,21,22,23]. Alongside these, other emerging technique such as centrifugal jet spinning, plasma-induced synthesis, and solution blow spinning (SBS) were also recently reported for the obtainment of nanofibrous mats [24,25]. Among these latter technologies, SBS is particularly interesting because of the possibility of producing nanofibrous membranes in short times and without a high energy consumption since it uses only a pressure air flow to promote the production of nanofibers. Therefore, a fast processing time and overall low costs could make the nanofibrous membranes obtained by SBS competitive and industrially scalable. SBS, however, presents some limits in regard to drugs encapsulation within nanofibers. In particular, during nanofibers production, liquid and solid drugs both emerge on the fibers surface due to the high evaporation rate of the solvent during the process [24,26], thus preventing the possibility of slowing down the release kinetics and obtaining a sustained release over time [27,28,29]. Considering that the correct encapsulation of liquid and solid drugs is crucial for achieving their controlled release, but also that appropriate production time and cost are essential for industrial scalability, it could be an interesting challenge to evaluate the existing difference, to form the production to the application, of the same drug delivery systems produced for ES or SBS.

Driven by environmental and health concerns, biodegradable polymers are replacing traditional polymers in many fields of application thanks to their low environmental impact and non-toxicity. One of the most prominent polymers among these is poly (lactic acid) (PLA), a biodegradable, thermoplastic, aliphatic polyester derived from renewable resources, such as corn starch or sugarcanes [30].

Chlorhexidine (CHX) is a hydrophilic antiseptic in powder form often used in the medical, dental, and pharmaceutical fields. It is effectively employed as a disinfectant and preservative due to its antibacterial properties towards both Gram-positive and Gram-negative bacterial strains [4]. Carvacrol (CRV) is a lipophilic essential oil obtained from oregano, an aromatic compound belonging to monoterpenoids. The latter has attracted much attention in food packaging applications owing to its unique antimicrobial, anticancer, and antioxidant activities [31].

The incorporation of nanosized particles into the matrix is one of the most successful strategies to enhance the performance of biopolymers [9,29,32,33]. Moreover, it was demonstrated that the presence of nanoparticles in the polymeric matrix could tune the drug release of those devices [34,35,36]. Among these, graphene nanoplatelets (GNP), belonging to the family of nanocarbons, are commonly used as fillers in polymer-based nanocomposites, aiming to enhance the mechanical and release performance [4,35,36,37,38,39,40].

In this work, a comparative study was performed on nanofibrous PLA-based systems for a controlled drug delivery application prepared with two different process techniques: ES and SBS. CRV was added to the polymeric solution as a liquid model molecule and, separately, CHX was added as a solid model molecule. In some cases, GNP were also added in order to study their effect on the release rate. Morphological, mechanical, and surface characterization were performed in order to study the effect of the two different production techniques on the obtained membranes and to test if with SBS it is possible to obtain devices suitable for drug delivery applications. Peppas–Korsmeyer mathematical model was applied to model the obtained data.

## 2. Results

The rheological analysis of the solutions was performed to evaluate the effects of model molecules and nanofillers on the rheological behavior of PLA solutions and, therefore, on their potential spinnability.

**Figure 1:**
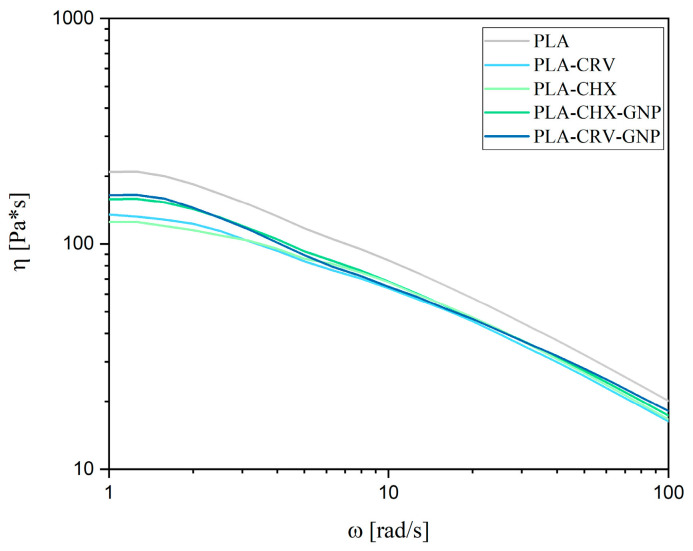
Complex viscosity of the polymeric solutions.

Figure 1 shows rheological analysis of neat PLA, PLA-CRV, PLA-CHX, PLA-CRV-GNP, and PLA-CHX-GNP solutions used for electrospinning and solution blow spinning.

All the systems displayed a Newtonian behavior in the low frequencies region and a remarkable shear thinning at higher frequencies. More in detail, in the low frequencies regions, the Newtonian viscosity values are higher for neat PLA if compared with all the other systems. As expected, adding either CRV or CHX induced a decrease in the viscosity values of the solutions, showing the lowest viscosity values of the whole set of samples [13]. This reduction, moreover, was already observed for similar systems when either CHX or [1] CRV [41] were added. On the other hand, GNP leads to an increase in viscosity at low frequencies for both PLA/CRV or PLA/CHX solutions, as already reported for other similar systems [1,42]. However, all the solutions were deemed to be compatible with the electrospinning process [15,37,43,44].

**Figure 2:**
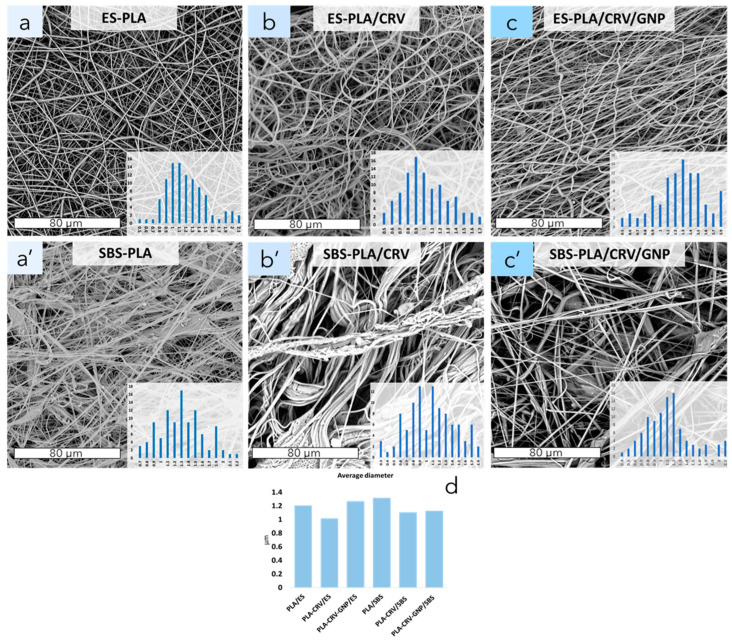
SEM micrographs of PLA nanofibers membranes obtained by electrospinning or solution blow spinning of ES-PLA, ES-PLA/CRV, ES-PLA/CRV/GNP (**a**–**c**) SBS-PLA, SBS-PLA/CRV, SBS-PLA/CRV/GNP (**a’**–**c’**), and relative averages diameters (**d**).

Figure 2a–c,a’–c’ shows the morphologies of the different nanofibrous membranes prepared by ES and SBS, respectively. While in Figure 2d, the average diameters of all the systems are reported, respectively. In ES-PLA (Figure 2a), fibers appear wavy and randomly oriented, showing a smooth and homogeneous diameter. Diameters are in the nanoscale range and show a unimodal distribution (inset of Figure 2a). When CRV is added to electrospun systems (Figure 2b), fibers appear wavier if compared to ES neat ones, and a decrease in the average diameter can be noted (Figure 2d). In fact, the addition of carvacrol, which acts as a plasticizer, causes a decrease in the polymeric solution viscosity, leading to the formation of thinner fibers [13,41,42,43,44,45].

When GNP and CRV are both added, for electrospun systems (Figure 2c), it is possible to notice more oriented fibers and a unimodal fibers diameter distribution. Moreover, an increase in the fibers’ average diameters can be also observed (Figure 2d). 

In regard to SBS systems (Figure 2a’–c’), SBS-PLA (Figure 2a’) shows straight fibers with some surface defects. Even in this case, the fiber diameters are in the nanoscale range, but a multimodal distribution can be observed if compared to ES-PLA fibers (subpanel of Figure 2a’). The addition of CRV (Figure 2b’) induced the formation of cohesive fiber bundles with an increase in their diameters (Figure 2d). Additionally, in this case, a multimodal distribution can be observed (subpanel of Figure 2b’). SBS-PLA/CRV/GNP (Figure 2c’) shows straight fibers with a unimodal size distribution and an increase in their average diameter can be observed (Figure 2d). It is known in the scientific literature that the addition of GNP promotes an increase in the solution viscosity [1,46] and conductivity [40], inducing an increase in the fiber diameter in SBS systems.

**Figure 3:**
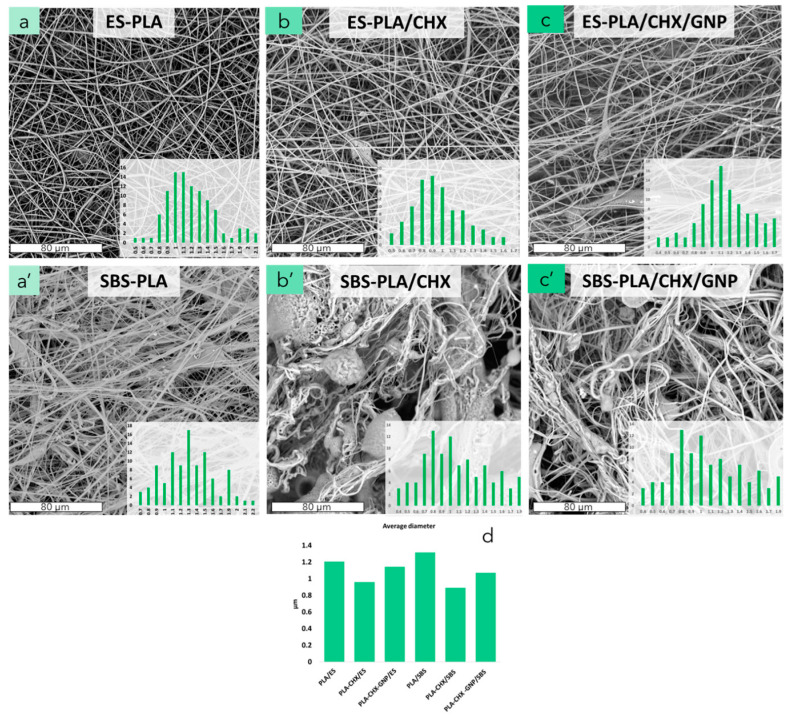
SEM micrographs of PLA nanofibers membranes obtained by electrospinning or solution blow spinning of ES-PLA, SBS-PLA, ES-PLA/CHX, SBS-PLA/CHX, ES-PLA/CHX/GNP, SBS-PLA/CHX/GNP (**a**–**c**,**a**’–**c**’), and relative averages diameters (**d**).

Figure 3 shows the morphologies of CHX containing nanofibrous membranes prepared by ES and SBS. ES-PLA and SBS-PLA (Figure 3a,a’) were again reported for comparison. ES-PLA/CHX (Figure 3b) shows randomly oriented, smooth, and homogeneous fibers with a unimodal fibers size distribution. Moreover, the presence of some beads can be noted. When GNP and CHX are either added in the ES systems (Figure 3c), it is possible to notice more oriented fibers and a unimodal fibers diameter distribution with an increase in the fiber diameter average if compared to ES-PLA/CHX (Figure 3d).

In regard to SBS systems, on the contrary, the addition of CHX (Figure 3b’), induced fiber bundles, and the contextual formation of large beads with a decrease in the fibers diameter (Figure 3b’) is due to a significant decrease in the relative solution viscosity. Furthermore, a multimodal distribution can be observed (inset of Figure 3b’). SBS-PLA/CHX/GNP (Figure 3c’) shows fibers bundles with a lower presence of beads, if compared to SBS-PLA/CHX, and a multimodal size distribution can be observed (inset of Figure 3c’). Additionally, in this case, the presence of GNP promotes an increase in the solutions viscosity, leading to the formation of more homogeneous fibers. An increase in the fiber diameter average can be also observed (Figure 3d).

The existing morphology differences between ES and SBS membranes can be explained considering that the polymeric solution is ejected from a capillary under a strong electrostatic force. Subsequently, the solvent evaporates and forms ultrafine wavy fibers on the collector. When SBS is adopted, instead, a high-velocity gas flow provides the driving force for the fabrication of nanofibers [24]. The pressurized high-velocity gas causes pressure drops and shearing at the gas/solution interface, resulting in stretching of the polymeric solution towards the fixed collector. As the solvent speedily evaporates, straight fibers are rapidly formed (see Figure 3a’,c’). 

**Figure 4:**
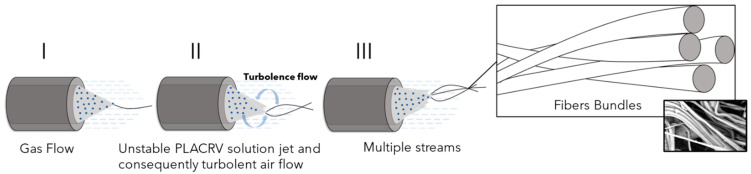
Pictorial representation of the formation of fibers bundles during SBS process.

However, when a solution with a low viscosity value is spun for SBS, a turbulent polymeric flow is generated, causing bending instability and, consequently, multiple streams of solution emerge from a single nozzle [24] (Figure 4). This behavior leads to the formation of fiber bundles, as can be noted in SBS-PLA/CRV, SBS-PLA/CRV/GNP, and SBS-PLA/CHX/GNP, SBS-PLA/CHX (see Figure 2b’,c’ and Figure 3b’,c’).

**Figure 5:**
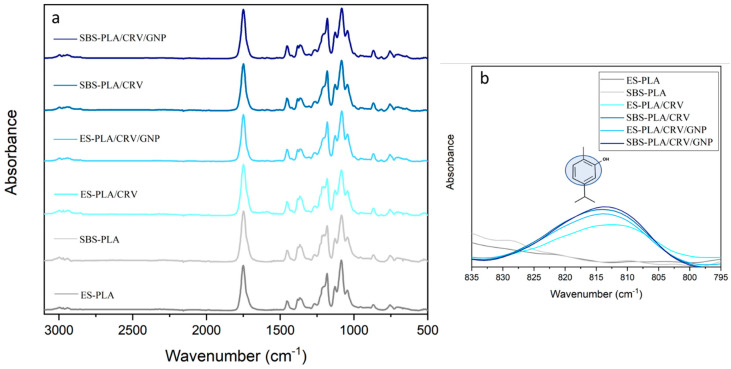
ATR-FTIR spectra of the CRV fibrous membranes (**a**) and a close-up of spectral range: 835–795 cm^−1^, relative to the peak at 1084/1091 cm^−1^ of CRV aromatic ring (**b**).

FTIR/ATR analysis was carried out with the purpose of detecting the effective presence of CRV in the nanofibrous membranes. The FTIR spectra collected in the range 3200–500 cm^−1^ is shown in Figure 5a. All the samples display the characteristic bands of PLA, such as those centered at 1750 cm^−1^ and 1150–1000 cm^−1^, respectively, referring to C=O and C-O stretching. It is possible to recognize the presence of CRV by monitoring the band centered at 814 cm^−1^, which is ascribed to the CRV aromatic ring [14]. The magnification of this spectral region is provided in Figure 5b. Notably, the absorbance of such a band was found to be more intense in SBS-PLA/CRV/GNP and SBS-PLA/CRV, while decreasing for ES-PLA/CRV/GNP and ES-PLA/CRV. The lowest signal is shown by ES-PLA/CRV. As expected, no characteristic peak of CRV could be detected in ES-PLA and SBS-PLA. 

**Figure 6:**
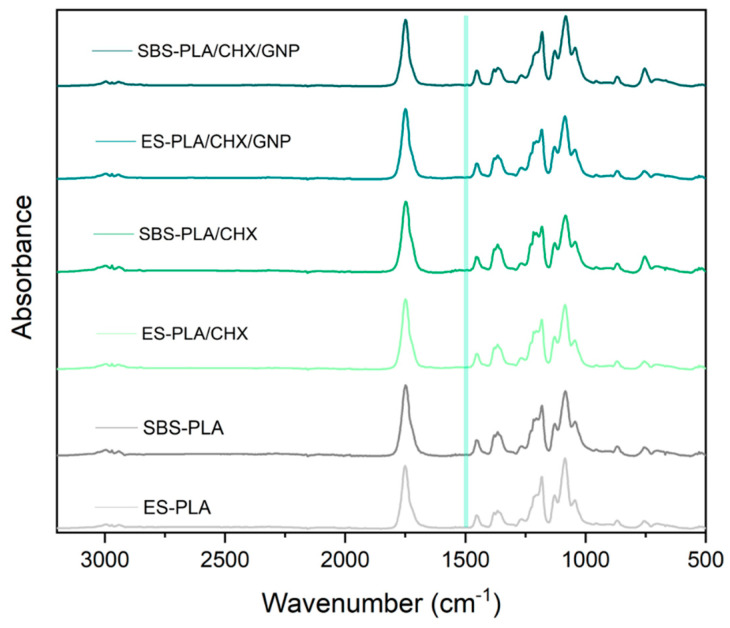
ATR-FTIR spectra of the CHX fibrous membranes.

Additionally, for CHX-containing systems, an ATR-FTIR analysis was performed on the surfaces of the membranes. The FTIR spectra collected in the range 3200–500 cm^−1^ is shown in Figure 6. All the samples display the characteristic bands of PLA as already described above. However, the characteristic peak of CHX (1490 cm^−1^) [47] could not be easily detected, as has already been reported for other similar system containing CHX [1].

**Figure 7:**
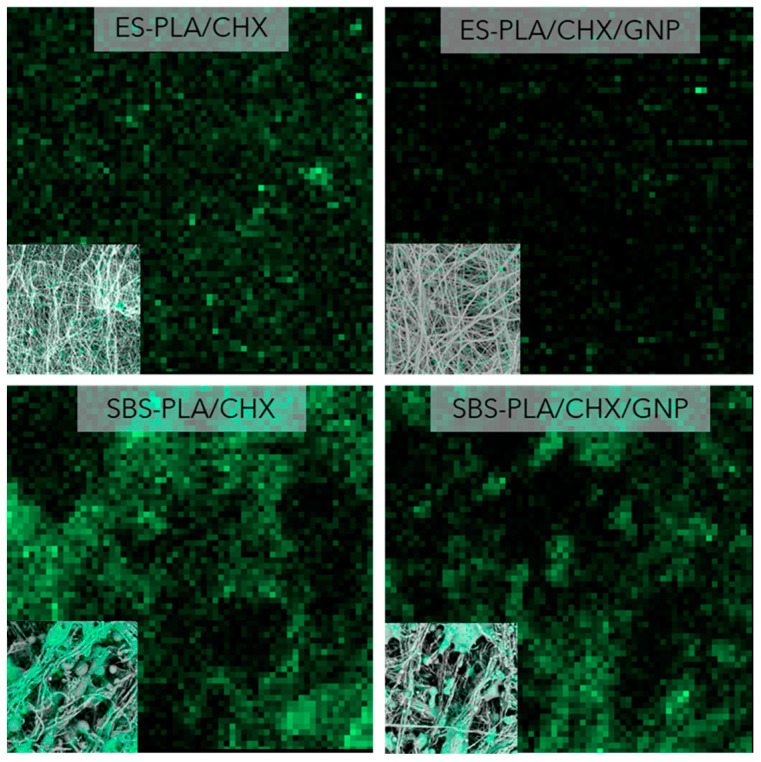
SEM-EDS analysis of nanofibers membranes obtained by electrospinning or solution blow spinning of ES-PLA/CHX, SBS-PLA/CHX, ES-PLA/CHX/GNP, SBS-PLA/CHX/GNP.

Aiming to confirm the effective presence of CHX, EDX analysis was performed on ES and SBS membranes to decipher the possible presence of the Cl signal, and the relative results are reported in Figure 7. SEM-EDS analysis confirmed that Chlorhexidine was correctly incorporated into the produced nanofibers. A high content of Cl can be noted on the surface of all CHX-filled samples. SBS systems transmitted a more intense signal of Cl compared to ES ones.

**Figure 8:**
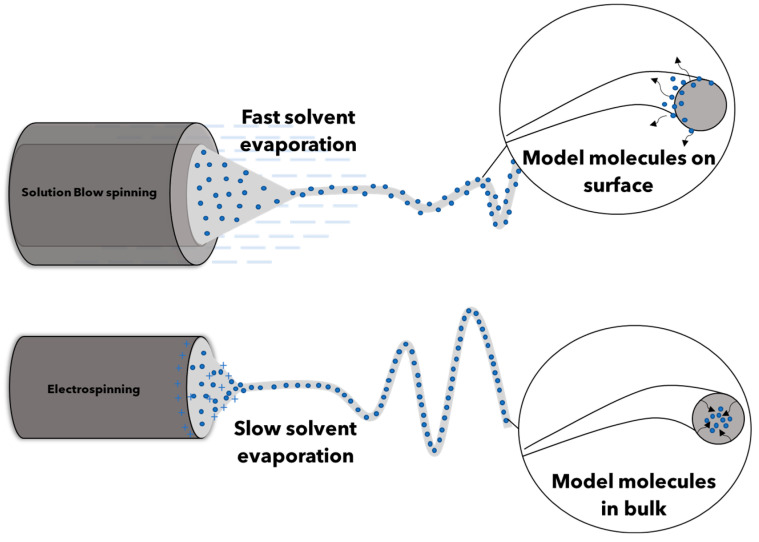
Pictorial representation of incorporation in SBS and ES processes.

Therefore, surface characterization displayed that both CRV and CHX showed a more intense signal in SBS systems if compared to ES ones. This behavior could be reasonably explained considering that, in general, SBS processing facilitates the migration of incorporated molecules on the fibers surface due to the fast evaporation of the solvent [24,25,26]. As is possible to notice from the graphical description in Figure 8, the high velocity air flow of SBS induces a fast evaporation of the solvent, allowing the molecule models to move on the fibers’ surface. On the contrary, in ES, the molecules are well embedded in the innermost part of the fibers. 

The mechanical properties of nanofibrous membranes were investigated by tensile tests and the values of the elastic modulus (E), tensile strength (TS), and elongation at the break (EB) are reported in Table 1. In detail, for neat systems, ES membranes showed an E value of 14.9 MPa and a TS of 2.8 MPa, while, when the same solution is spun for SBS, the elastic modulus increased to 25.8 MPa and the TS decreased to 2.1 MPa. Moreover, PLA/SBS membranes showed a lower elongational at the break compared to the ES one. This behavior could be reasonably ascribed to the already observed differences in the membranes’ morphology. The addition of CRV leads to different behaviors in ES and SBS systems. PLA-CRV/ES showed an increase in ductility (increasing from 425 MPa to 845.9 MPa) and in E if compared with neat PLA/ES. This behavior could be reasonably ascribed to the plasticizing effect imparted by CRV [13,48,49]. Additionally, PLA-CRV/SBS displayed an increase in the E value, increasing from 25.8 MPa of the neat system to 32.5 MPa probably due to the presence of fiber bundles in the morphology of the PLA-CRV/SBS membranes. The addition of GNP, in SBS and ES CRV systems, leads to an increase in the E value. In fact, it is known in the scientific literature that by adding graphene nanoplatelets, it is possible to improve the mechanical performance of the nanofibrous mat [33,39,50].

It was observed that a CHX addition did not lead to any significant variation in the mechanical performance of ES-PLA/CHX systems. On the contrary, in SBS-PLA/CHX, a decrease in the E and TS and an increase in the membrane ductility could be noted. This behavior is probably due to the high presence of beads in the morphology of the membrane (in agreement with the morphological characterizations see Figure 3b’) and to the plasticizing effect provided by the drug, as already confirmed by rheological tests. When CHX and GNP are both present in the ES system, an increase in the E (increasing from 14.9 to 87 MPa) and a decrease in the EB (decreasing from 425 to 200%) can be noted compared to the neat ES-PLA.

On the other hand, the presence of GNP in SBS-PLA/CHX/GNP improves the mechanical performance, with the E, TS, and EB value increasing from 25 MPa, 2.1 MPa, and 21% of the neat membrane to 55.8 MPa, 2 MPa, and 153%, respectively.

It could be generally concluded that in all systems in which fiber bundles are present, an increase in the mechanical properties could be noted.

**Figure 9:**
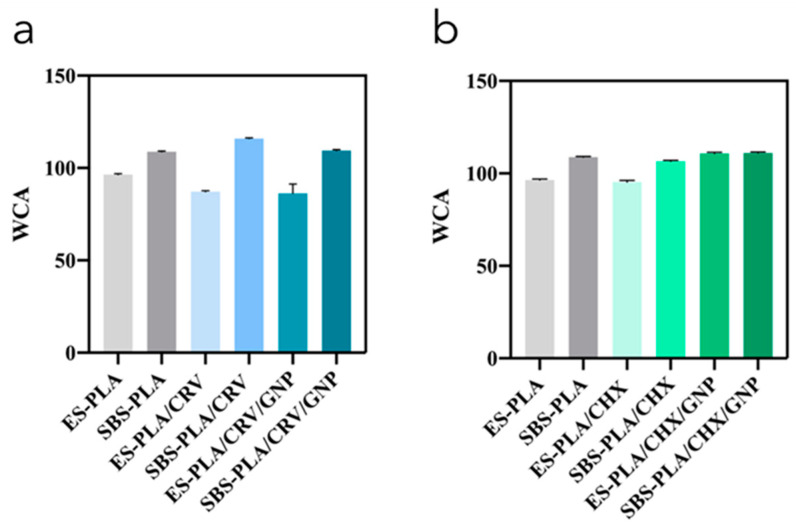
Water contact angles of ES and SBS nanofiber mats filled with CRV (**a**) and CHX (**b**).

Figure 9a,b shows WCA values of different CHX- or CRV-containing nanofibrous membranes. The neat systems prepared by ES and SBS show the typical hydrophobic behavior of PLA-based nanofibrous membranes [51] displaying water contact angles of 96° and 108°, respectively. The increase in the WCA values of PLA/SBS, if compared to the ES counterpart, could be reasonably ascribed to the increase in the surface roughness due to the defects present on the SBS-PLA membranes’ surface (see Figure 2a). 

In regard to CRV-containing systems, the addition of it to electrospun membranes induces a decrease in the PLA hydrophobic behavior [13,51]. In fact, SBS-PLA/CRV shows a contact angle of 87°. Moreover, as predicted, by adding GNP to the systems, a decrease in the wettability is reported [34] with WCA values decreasing from 96° of neat PLA to 87° and 86° of ES-PLA/CRV and ES-PLA/CRV/GNP, respectively. SBS systems, instead, show a higher hydrophobic behavior [52]: SBS-PLA, SBS-PLA/CRV, and SBS-PLA/CRV/GNP show WCA values of 108°, 113°, and 109°, respectively. These increases in the WCA value could be reasonably explained considering the morphological characterizations of the membrane’s surfaces; in particular, SBS-PLA/CRV presents fiber bundles which probably induce an increase in the surface roughness and, consequently, an increase in the surface wettability according to the Wetzel equation [6]. The addition of the nanofillers, however, promoted the formation of more homogeneous and detached fibers, leading to WCA values comparable to pure ones.

WCA values of different CHX-containing nanofibrous membranes systems are shown in Figure 9b. The presence of CHX induces a decrease in WCA values for both processing techniques, with contact angles ranging from 96° and 109° of ES-PLA and SBS-PLA to 95° and 106° of ES-PLA/CHX and SBS-PLA/CHX, respectively. Additionally, in this case, the addition of GNP induces a decrease in the wettability for both systems; in fact, SBS-PLA/CHX/GNP and ES-PLA/CHX/GNP show WCA values of 109° and 118°, respectively.

**Figure 10:**
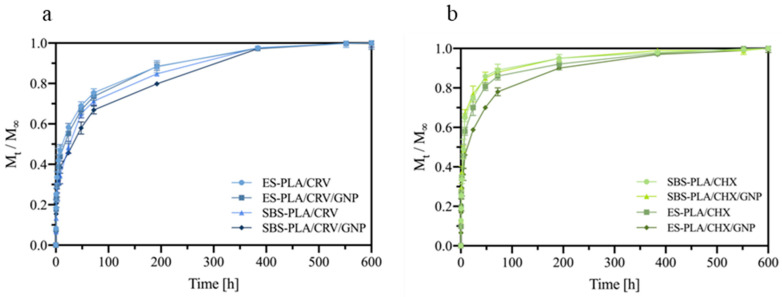
Release kinetics of CRV (**a**) and CHX (**b**) from membranes in deionized water at 37 °C expressed as M_t_/M_∞_. Each measurement was performed in triplicate.

The release kinetics of CRV and CHX are shown in Figure 10a,b, respectively, as the ratio of M_t_/M_∞_, where M_t_ is the amount of drug released at time t, and M_∞_ is the theoretical amount of CRV or CHX incorporated in the membranes. For all systems, the release of CHX or CRV is characterized by at least three phases: a burst release at the early stage of the immersion time, a second phase characterized by the progressive depletion of the molecules model at a slower release rate, and a final plateau region after long-term immersion. 

In regard to the CRV release (Figure 10a), ES-PLA/CRV and ES-PLA/CRV/GNP release about 50% in the first 5 h and reach the plateau region after about 500 h. On the other hand, SBS-PLA/CRV show a more sustained release, in which about 40% of CRV is released in the first 5 h, reaching the plateau region after 500 h. The same behavior can be noted for SBS-PLA/CRV/GNP; moreover; the presence of GNP promoted a more effective sustained release, as has already been reported for other systems [1,13]. SBS systems allow for obtaining a more sustained release due to their further hydrophobic behavior compared to systems made by ES, according to surface wettability tests (see Figure 9a). 

Systems containing CHX (Figure 10b) show the opposite behavior. In detail, ES-PLA/CHX and ES-PLA/CHX/GNP release about 60% of CHX in the first 5 h and reach the plateau region after about 500 h. SBS-PLA/CHX and SBS-PLA/CHX/GNP, instead, show a more remarkable burst release, with 70% of CHX released in the first 5 h, followed by a sustained release until the plateau region is reached. In fact, according to the EDX characterizations, a high amount of CHX is available in the SBS fibers surface due to the rapid evaporation of the solvent during the process. Therefore, most of CHX is instantaneously released when membranes are submerged in water. On the contrary, CHX molecules are well embedded in ES fibers. This behavior leads to a more sustained release for ES systems. This testifies again that the ES technique is more appropriate for incorporating solid molecules inside the fibers if compared to a solution blow the spinning ones. Therefore, the ES technique should be preferred if a more sustainable release of CHX is mandatory. 

To confirm these hypotheses on the CRV and CHX release mechanism, the obtained release data were fit according to Peppas–Korsmeyer mathematical model [14,53]. Moreover, this model has previously been successfully used to describe drug release from nanofibers [1]. The value of k and n of the different formulations were determined and reported in Table 2.

**Figure 11:**
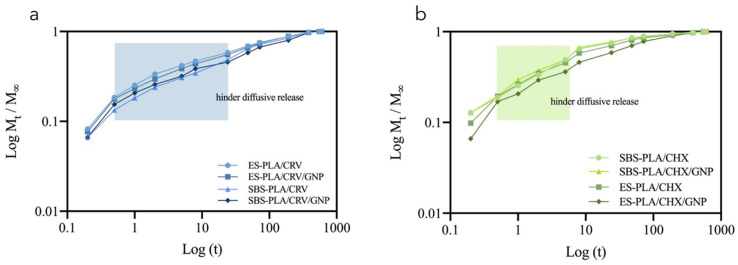
Logarithmic plots of power law model applied to the release data collected in the region M_t_/M_∞_ < 0.6 for all CRV (**a**) and CHX (**b**) containing systems.

The logarithmic plots of CRV and CHX release data as a function of time (Figure 11a,b, respectively), which were evaluated to further understand the drugs release mechanism. In the first 30 min of the release test, all devices showed a burst release region in which CRV or CHX molecules, available on the fibers’ surface, are immediately delivered in the aqueous medium. Subsequently, the release seems to be driven by diffusive phenomena for all systems.

Through the n value, it is possible to obtain information about the release mechanism. In fact, when n is approximately 0.5, the fertilizer delivery is driven by diffusive phenomena, and *n* values between 0.5 and 1 indicate an anomalous release [53]. However, the biopolymeric matrix may hinder diffusion, thus n<0.5. 

In particular, all CRV systems (Figure 11a) showed a burst region characterized by faster kinetics, probably attributable to some CRV molecules available in the fibers’ surface and thus making them faster delivered, and a second region that provide information about the actual capability of the system to establish a controlled release characterized by a hindered diffusion (n=0.31). All CHX systems (Figure 11b) showed the same scenario with a first region characterized by faster kinetics and a second region characterized by a hinder diffusion of CHX (n=0.38,0.41,0.46,0.41), ensuring an almost Fickian release mechanism. 

The inhibitory properties of the nanofibers’ membranes developed with the two processes SBS and ES in this study were evaluated in vitro against four main pathogenic bacterial species, including two Gram-positive (*L. monocytogenes* and *S. aureus*) and two Gram-negative (*E. coli* and *P. aeruginosa*), which are known agents of food-borne diseases [54]. The results of the antimicrobial assay are reported in Table 3. In general, considering that the diameter of the inhibition areas generated by the nanofibrous discs developed with the two techniques (ranging from 7 to 28 mm), Gram-negative bacteria were less sensitive than *L. monocytogenes* ATCC 19114 and *S. aureus* ATCC 33862, even though *E. coli* ATCC 25922 resulted in being inhibited by nine nanofibrous discs, while both Gram-positive indicators were inhibited by eight discs. *P. aeruginosa* ATCC 27853 was inhibited, indeed at a very low sensitive level, only by the discs obtained from SBS; in particular, SBS-PLA/CRV/GNP and SBS-PLA/CHX. Thus, the sensitivity of this strain was quite negligible. Undoubtedly, the highest inhibitory activity was registered in the presence of discs processed by SBS technology; SBS-PLA/CRV/GNP inhibited all four indicator strains, showing a strong power, especially against *L. monocytogenes* ATCC 19114 (28 mm width). This species has been long considered an emerging pathogen, but due to the high fatality rate exceeding 30% [55], it is now well recognized as a human pathogen. Various strains of this species resulted in being highly sensitive to several essential oils and their edible film formulations [56]. SBS-PLA/CRV/GNP also determined a consistent inhibition of *E. coli* ATCC 25922 and *S. aureus* ATCC 33862 (22 and 21 mm diameter of the inhibition area, respectively). Although the high sensitivity of Gram-positive bacteria is easily explained by the absence of the outer membrane in the cell wall layer [57], the data registered for *E. coli* ATCC 25922 are quite interesting. However, the effectiveness of carvacrol against *E. coli*, even in micelle-encapsulated form, is known [58]. Despite the different susceptibilities to various essential oils displayed by Gram-positive and Gram-negative bacteria [59], in the case of *E. coli*, carvacrol determines a non-specific permeabilization of the cytoplasmic membrane with the consequent release of ATP from cells. Furthermore, the treatment of *E. coli* with carvacrol is also consistent with cytoplasmic membrane disruption [60]. 

Regarding ES membranes, they were much less effective than SBS discs against the same pathogens. *P. aeruginosa* ATCC 27853 was not inhibited at all. *L. monocytogenes* ATCC 19114 and *S. aureus* ATCC 33862 were slightly inhibited by ES-PLA/GNP ES-PLA/CRV, ES-PLA/CHX and ES-PLA/CHX/GNP, and ES trials (7–10 mm width of clear halos), while *E. coli* ATCC 25922 showed a sensitivity also to ES-PLA/CRV, even at a very low level (8 mm width). 

## 3. Materials and Method

### 3.1. Materials

The raw materials used in this work were poly lactic acid (PLA) (Natureworks, 2003D grade), density = 1.25 g/cm^3^, melt flow index = 6 g/10 min, content of D-lactic acid monomer 4.3%), purchased from NatureWorks. A liquid sample of carvacrol (CRV, (2-methyl-5-(1-methylethyl)-phenol, density = 0.977 g/L, purity ≥ 97%), chlorhexidine (CHX), chloroform (CF), and acetone (Ac) were purchased from Sigma Aldrich. The saturated solubilities of carvacrol and chlorhexidine are 0.33 g/L and 0.19 g/L, respectively. Graphene nanoplatelets (GNP), trade name xGnP^®^, Grade C, were supplied by XG Sciences Inc., Lansing, MI, USA. Each particle consists of several sheets of graphene with an average thickness of approximately 10–20 nm, average diameter between 1 and 2 μm, and a specific surface area of about 750 m^2^/g. All the reactants were used as received.

### 3.2. Preparation

In total, 10% (*w/v*) of PLA solution was prepared by dissolving PLA in a CF/Ac mixture (2:1 ratio) under magnetic stirring at 25 °C overnight. In order to test the membranes release ability, either CHX or CRV were added to the polymeric solution at 5 wt% and 14 wt%, respectively (respect to the polymer concentration), whereas for preparing the (nano) composite mats, GNP at 1 wt% with respect to the polymeric solution was added to the above polymeric solution.

The PLA-based membranes were prepared by adopting two different kinds of processing, namely, electrospinning (ES) and solution blow spinning (SBS).

In order to prepare ES membranes, each polymeric solution was poured into a 10 mL plastic syringe equipped with a 16 g metal needle and were electrospun onto a grounded collector wrapped in an aluminum foil, by using a conventional electrospinning equipment (Linari Engineering-Biomedical Division, Pisa, Italy). The following conditions were kept constants for all the samples: feed rate = 3 mL/h, voltage applied = 15 kV, needle-to-collector distance = 15 cm, electrospinning time = 2 h. 

The SBS apparatus consisted in a source of compressed air, equipped with a pressure regulator, a 10 mL glass syringe, a syringe pump to control the injection rate of the polymer solutions and a spinning apparatus that consisted of a setup with concentric nozzles, and a static collector wrapped in aluminum foil. The following operating conditions were kept constant for all the samples: feed rate = 50 mL/h, pressure applied = 0.6 MPa, needle-to-collector distance = 15 cm, solution blow spinning time = 5 min. 

**Figure 12:**
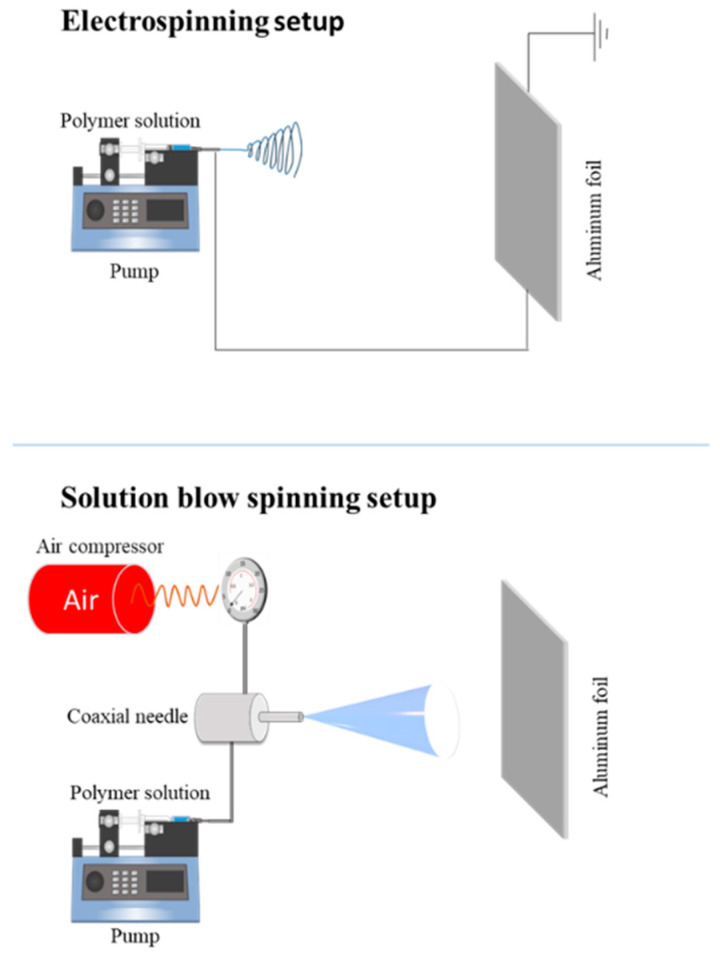
Electrospinning and solution blow spinning setup.

Figure 12 provides a pictorial description of the two different routes adopted for producing different membranes, while in Table 4, the sample codes name of the systems and their related formulations are reported.

### 3.3. Morphological Analysis

In order to compare the different morphology of the nanofibers obtained by ES or SBS, a morphological analysis of the membranes was analyzed by using a scanning electron microscope (Phenom ProX, Phenom-World, the (Eindhoven, The Netherlands) with an optical magnification range of 20–135x, electron magnification range of 80–130,000×, maximal digital zoom of 12×, and an acceleration voltage of 15 kV. The microscope was equipped with a temperature controlled (25 °C) sample holder. The samples were positioned on an aluminum stub using an adhesive carbon tape. The elemental mapping, focusing on Cl, was analyzed using an energy dispersive X-ray analyzer EDX integrated with scanning electron microscopy. An open-source image processing software (Image J) equipped with a plug-in (Diameter J) was used to evaluate the diameter size distribution and mean size of the fibers.

### 3.4. Rheological Characterization

The rheological behavior of the solutions plays a central role in determining their spinnability. Considering that the rheological properties of all the polymeric solutions were measured, we used a rotational rheometer (ARES-G2) equipped with a 25 mm parallel-plate geometry. All tests were performed at 25 °C in frequency sweep mode in the range 1–100 rad/s by imposing a constant stress of 1 Pa.

### 3.5. FT-IR/ATR Analysis

The chemical and structural characterization of the membranes were assessed by FT-IR/ATR analysis, carried out by using a Perkin–Elmer FT-IR/NIR Spectrum 400 spectrophotometer. The spectra were recorded in the wavenumber range 4000–400 cm^−1^.

### 3.6. Mechanical Properties

The mechanical behavior of the membranes was investigated by tensile tests, carried out with a laboratory dynamometer (Instron model 3365, UK) equipped with a 1 kN load cell. The tests were performed on rectangular shaped specimens (10 × 90 mm) cut off directly from the membranes. The measurements were performed by using a double crosshead speed: 1 mm min^−1^ for 2 min and 50 mm min^−1^ until fracture occurred. The grip distance was 30 mm, whereas the sample thickness was measured before each test. Eight specimens were tested for each sample and the outcomes of the elastic modulus (E), tensile strength (TS), and elongation at break (EB) have been reported as average values ± standard deviations. 

### 3.7. Water Contact Angle (WCA) Measurements 

Surface wettability of ES and SBS membranes was determined by WCA testing (First Ten Angstroms FTA 1000, Cambridge, UK). An automatic liquid drop dosing system drips 4 μL of distilled water onto the samples surfaces and after 20 s, images were taken. Five different spots of each sample were tested and WCA values have been reported as average values ± standard deviations.

### 3.8. Release Tests

A series of DI water solutions containing a known amount of CHX or CRV were analyzed by using a UV–Vis spectrophotometer (model UVPC 2401, Shimadzu Italia s.r.l., Milan, Italy) in order to obtain a calibration line to correlate the absorbance band intensity of CHX or CRV and its concentration in DI water. The maximum absorbance band of CHX was detected at 230 nm and the maximum absorbance of CRV was detected at 273 nm [13]. The release of the antiseptic or the antimicrobial from the membranes was investigated by immersing pre-weighed square specimens (10 × 10 mm^2^, 0.01 g ca.) in 10 mL of DI water at 37 °C. The absorbance band intensity of the storage solutions was measured at specific time intervals and converted to CHX or CRV released through the calibration line. All the samples were immersed in 10 mL of fresh DI water after each measurement. Each measurement was performed in triplicate.

### 3.9. Antimicrobial Assay

The strains Escherichia coli ATCC 25922, Listeria monocytogenes ATCC 19114, Pseudomonas aeruginosa ATCC 27853, and Staphylococcus Aureus ATCC 33862 were all provided by the American Type Culture Collection and tested in this study as indicator organisms, sensitive to antimicrobial compounds. All strains were reactivated overnight in the optimal growth conditions: *P. aeruginosa* ATCC 27853 in nutrient broth (NB) (Oxoid, Milan, Italy) at 25 °C, while all other strains in brain heart infusion (BHI) broth (Oxoid) at 37 °C. 

The inhibitory test was conducted applying the principle of paper disc diffusion. A double agar layer was prepared pouring water agar (2% *w/v*) as the bottom layer and BHI or NB soft agar (0.7% *w/v*), inoculated with approximately 107 CFU mL^−1^ of each indicator strain, as a top layer with a 9 cm diameter in Petri dishes. Discs of a 6 mm diameter were cut from each sample with a sterile steel cork borer and placed onto the double agar layer at an approximate distance of 3 cm from one another. A 6 mm diameter Whatman No. 1 filter was soaked in streptomycin solution (10% *w/v*) and used as the positive control. Before incubation, all plates were kept refrigerated for 2 h to allow the radial diffusion of the active compounds from the discs; the incubation occurred at the optimal temperature for each strain for 24 h. The results were deemed to be positive if a clear area was observed around the discs and the width of the inhibition halos was measured.

## 4. Conclusions

A comparative study was performed on nanofibrous PLA-based systems for controlled drug delivery application prepared with two different process technique: electrospinning (ES) and solution blow spinning (SBS). In general, both techniques allow for obtaining nanometric fibers characterized by a similar mechanical performance. In order to test the release ability of these systems, carvacrol (CRV) was added to the polymeric solution as a liquid model molecule, and chlorhexidine (CHX) was selected as the solid one. Both molecules were correctly embedded in the nanofibers. However, ES leads to the incorporation of CRV and CHX in a more inner part of the fibers compared to SBS ones, in which the molecules are placed on the fibers’ surface, as also confirmed by FTIR and EDS analysis. Moreover, the outcomes reveal that the morphology, surface characteristics, and, consequently, also the release kinetics of the membranes were affected by solution composition and process technique. SBS allows for obtaining membranes with a higher WCA value compared with their ES counterpart, effecting their release kinetics. In detail, the lower wettability of the SBS system can be reasonably ascribed to their morphology, characterized by the presence of fiber bundles. This peculiar morphology allows for a slowing down of the CRV release kinetics compared to the ones obtained for ES membranes. On the contrary, with SBS, a faster CHX release can be obtained due to its hydrophilic behavior. In SBS, in fact, CHX molecules, being laid out on the surface of the fibers, rapidly dissolve when the membrane is submerged in DI water. Moreover, GNP addition, leading to a decrease in wettability, allowed for a slowing down of the release kinetics in whole systems.

## Figures and Tables

**Table 1:** Elastic modulus (E), tensile strength (TS), and elongational break (EB) values of PLA, PLA-CRV, PLA-CRV-GNP, PLA-CHX, and PLA-CHX-GNP membranes obtained by ES and SBS processes.

Sample	E [MPa]	TS [MPa]	EB [%]
ES-PLA	14.9 ± 1.2	2.8 ± 0.2	425 ± 0.5
SBS-PLA	25.8 ± 0.3	2.1 ± 0.1	21.8 ± 3.1
ES-PLA/CRV	29.69 ± 0.7	2.5 ± 0.3	845.9 ± 6
SBS-PLA/CRV	32.5 ± 0.2	1.72 ± 0.2	147.8 ± 2.3
ES-PLA/CHX	16.8 ± 0.6	2.3 ± 0	428.9 ± 12
SBS-PLA/CHX	9.7 ± 1.2	0.5 ± 0.5	35.7 ± 0.8
ES-PLA/CRV/GNP	95.6 ± 2	2.8 ± 0.3	273.7 ± 2.5
SBS-PLA/CRV/GNP	135.2 ± 2.3	2.9 ± 0.8	154.6 ± 3.1
ES-PLA/CHX/GNP	87.14 ± 2	2.4 ± 1	200 ± 12
SBS-PLA/CHX/GNP	55.8 ± 0.9	2 ± 0.2	153.63 ± 2.1

**Table 2:** Values of slopes (n) and intercepts (k) of fitting of Peppas–Korsmeyer model power law applied to the release data collected in the burst region (M_t_/M_∞_ < 0.6).

Sample Code Name	*k*	*n*
ES-PLA/CRV	0.25	0.31
ES-PLA/CRV/GNP	0.23	0.31
SBS-PLA/CRV	0.18	0.31
SBS-PLA/CRV/GNP	0.20	0.31
ES-PLA/CHX	0.25	0.41
ES-PLA/CHX/GNP	0.25	0.38
SBS-PLA/CHX	0.27	0.46
SBS-PLA/CHX/GNP	0.19	0.41

**Table 3:** Antimicrobial assay values of SBS and ES nanofibrous membranes.

SAMPLE CODE	TYPE (Strains)			
	*Escherichia coli* (ATCC 25922)	*Pseudomonas aeruginosa* (ATCC 27853)	*Listeria monocytogenes* (ATCC 19114)	*Staphylococcus aureus* (ATCC 33862)
	Spot (mm)			
CP	28	26	43	30
SBS-PLA	-	-	-	-
SBS-PLA/CRV	15	-	12	8
SBS-PLA/CRV/GNP	22	7	28	21
SBS-PLA/CHX	10	7	14	11
SBS-PLA/CHX/GNP	12	-	10	14
ES-PLA	-	-	-	-
ES-PLA/CRV	8	-	-	-
ES-PLA/CRV/GNP	10	-	8	10
ES-PLA/CHX	7	-	7	10
ES-PLA/CHX/GNP	7	-	8	9

**Table 4:** Sample code name and formulations of the relative solutions.

Sample Code	Process Method	PLA (wt%)	CHX (wt%)	CRV (wt%)	GNP (wt%)
ES-PLA	Electrospinning	10	-	-	-
SBS-PLA	Solution blow spinning	10	-	-	-
ES-PLA/CRV	Electrospinning	10	-	14	-
SBS-PLA/CRV	Solution blow spinning	10	-	14	-
ES-PLA/CHX	Electrospinning	10	5	-	-
SBS-PLA/CHX	Solution blow spinning	10	5	-	-
ES-PLA/CRV/GNP	Electrospinning	10	-	14	1
SBS-PLA/CRV/GNP	Solution blow spinning	10	-	14	1
ES-PLA/CHX/GNP	Electrospinning	10	5	-	1
SBS-PLA/CHX/GNP	Solution blow spinning	10	5	-	1

## Data Availability

The rough/processed data that support our study are available from the corresponding author on reasonable request.

## Sample Availability

Samples of the compounds are not available from the authors.
